# From Protrusion to Extrusion: Reassessing the Selective Role of Intradiscal Interventions

**DOI:** 10.5812/aapm-168119

**Published:** 2026-02-28

**Authors:** Babak Eslami, Reza Atef Yekta, Hossein Majedi, Alireza Khajehnasiri, Ebrahim Espahbodi, Alireza Montaseri, Maziar Maghsoudloo, Noushin Rezaalizade, Alireza Rezaee

**Affiliations:** 1 Department of Anesthesiology, Intensive Care and Pain Medicine, Imam Khomeini Hospital Complex, Tehran University of Medical Sciences, Tehran, Iran; 2 Pain Research Center, Neuroscience Institute, Tehran University of Medical Sciences, Tehran, Iran; 3 Department of Anesthesiology, Intensive Care and Pain Medicine, Shariati Hospital, Tehran University of Medical Sciences, Tehran, Iran; 4 Alan Edwards Pain Management Unit, Department of Anesthesia, Montreal General Hospital, McGill University, Montreal, QC, Canada

**Keywords:** Lumbar Disc Herniation, Intradiscal Procedures, Percutaneous Laser Disc Decompression, Radiopaque Gelified Ethanol, Interventional Pain Medicine

## Abstract

**Context:**

Extruded lumbar disc herniations are traditionally managed surgically, whereas contained protrusions are more commonly considered suitable for percutaneous intradiscal procedures. However, extrusion itself may not be a universal contraindication to all needle-based intradiscal interventions, particularly when the fragment is non-sequestered and remains continuous with the parent disc. This narrative review evaluates the evidence, safety considerations, and clinical positioning of intradiscal procedures in extruded, non-sequestered lumbar disc herniations.

**Evidence Acquisition:**

A structured narrative review was conducted using PubMed/MEDLINE, Embase, Scopus, the Cochrane Library, Google Scholar, reference-list screening, and selected technical or manufacturer documents. The review focused on intradiscal interventions used or debated for lumbar disc herniation with radicular symptoms, including percutaneous laser disc decompression and targeted PLDD (T-PLDD), radiofrequency nucleoplasty, oxygen-ozone (O_2_-O_3_) discolysis, gelified ethanol, and mechanical decompression devices. Epidural injections, endoscopic or open discectomy, and procedures primarily investigated for discogenic axial low back pain or the regenerative treatment of degenerative disc disease were excluded from the main synthesis. Evidence was interpreted according to study design, disc morphology, direct applicability to extruded non-sequestered herniation, clinical outcomes, and safety reporting.

**Results:**

The evidence base is limited and heterogeneous. T-PLDD and radiofrequency nucleoplasty provide the most direct, although still low-certainty, clinical evidence for extruded or uncontained herniations. Oxygen-ozone discolysis has biological plausibility, particularly through modulation of inflammation and potential facilitation of fragment resorption; however, most clinical data are derived from mixed-morphology cohorts. Gelified ethanol has encouraging observational data, including studies that permitted non-contained lesions, but extrusion-specific outcomes remain insufficient. Mechanical decompression devices differ from the other modalities because the reviewed evidence and device technical guidance primarily relate to contained herniations, free fragments are excluded in the manufacturer's technical guide, and extrapolation to extruded or sequestered lesions is not supported.

**Conclusions:**

Extrusion alone should not be considered a universal contraindication to all intradiscal interventions. Nevertheless, current evidence remains insufficient to support these procedures as alternatives to guideline-supported conservative care or surgical discectomy. Intradiscal interventions may be considered only as selective, non-first-line, intermediate options in carefully selected patients with extruded but non-sequestered lumbar disc herniations, preserved continuity with the parent disc, no progressive neurological deficit, and appropriate counseling regarding uncertain benefit and the possibility of surgical crossover. High-quality, extrusion-specific studies using standardized morphology definitions and rigorous safety reporting are needed.

## 1. Context

Lumbar disc herniation is among the most common causes of radiculopathy and chronic low back pain and has a substantial socioeconomic impact worldwide. Among its morphological subtypes, distinguishing contained protrusions from extrusions has important clinical and therapeutic implications. However, definitions of extrusion vary considerably across the literature (1). Some classifications rely on loss of continuity of the annulus fibrosus, whereas others differentiate between "uncontained" discs and "sequestered fragments" or describe "mushroom-shaped" herniations as subcategories. This variability complicates clinical decision-making and the interpretation of outcomes in interventional studies (2).

Traditionally, guideline-based care for lumbar disc herniation with radiculopathy follows a conservative-first approach in the absence of red flags or urgent neurological indications. This approach generally includes patient education, activity modification, exercise-based rehabilitation or physical therapy, and analgesic or anti-inflammatory medication when appropriate (3-6). Epidural injection of local anesthetic and steroid may be considered in selected patients with acute severe sciatica, but this remains a perineural rather than an intradiscal intervention and is therefore outside the main scope of this review (5, 6). When non-surgical treatment fails to improve pain or function and imaging findings remain concordant with symptoms, surgical decompression or discectomy remains an established treatment option, particularly when disabling radicular pain persists or neurological compromise is present (4, 6, 7). Existing guidelines and guideline reviews provide limited direction regarding percutaneous, needle-based intradiscal procedures, which are not strongly endorsed as first-line treatments and are rarely addressed separately for extruded, non-sequestered discs (3-6).

In daily practice, an implicit dichotomy often arises: extruded discs are more readily referred for surgery, whereas protruded discs are considered candidates for minimally invasive intradiscal procedures. However, this division appears to reflect excellent surgical outcomes in extrusions and comparatively poorer surgical results in contained protrusions, rather than intrinsic contraindications to intradiscal therapies in extruded discs. Indeed, most trials and case series have grouped protrusions and extrusions together, and very few studies have examined the safety and efficacy of intradiscal techniques specifically in extruded, non-sequestered discs (8-10).

Further complexity arises from the diversity of intradiscal modalities. Since the introduction of percutaneous laser disc decompression (PLDD) by Choy in the 1980s, numerous minimally invasive options have emerged, including targeted PLDD (T-PLDD), radiofrequency nucleoplasty, ozone therapy, gelified ethanol injection, and mechanical decompression devices. Each differs in mechanism, candidate selection, and safety profile. The rapid evolution of these technologies makes it difficult to generalize about "intradiscal therapy" as a single category (8-13).

Given these uncertainties, this narrative review aims to synthesize the available evidence regarding intradiscal procedures specifically for extruded lumbar discs. We focus on safety, efficacy signals, and patient selection, with particular attention to non-sequestered extrusions in which continuity with the parent disc is preserved. By situating these techniques within the framework of established clinical guidelines, this review seeks to clarify whether needle-based intradiscal interventions may be considered selective, non-first-line intermediate options for carefully selected patients, rather than being viewed as categorically contraindicated solely because of extrusion.

Accurate terminology is critical when discussing lumbar disc pathology because herniation morphology determines both therapeutic options and expected outcomes. A bulge refers to a circumferential, symmetric extension of disc tissue beyond the vertebral margins and does not qualify as a focal herniation. A protrusion, often termed a contained herniation, indicates focal extension of the nucleus pulposus beyond the disc margin with preservation of the outer annulus and posterior longitudinal ligament; in at least one plane, the base of attachment is wider than the displaced portion (1). By contrast, an extrusion, or uncontained herniation, is characterized by focal extension through a defect in the annulus or posterior longitudinal ligament such that, in at least one plane, the displaced fragment's diameter exceeds the width of its neck of origin. Continuity with the parent disc may persist or be lost. When continuity is completely absent, the lesion is referred to as sequestration, often associated with cranial or caudal migration of the fragment (2).

For the purposes of this review, the term "extruded disc" refers specifically to uncontained but non-sequestered herniations in which the fragment remains in continuity with the parent disc. This distinction is essential because most intradiscal techniques are designed to target tissue that is within or immediately contiguous to the disc space, and fully sequestered fragments fall outside their intended mechanism of action.

A major challenge in interpreting the literature is the lack of uniform definitions. Some studies classify all uncontained herniations as extrusions, whereas others exclude sequestrations or use descriptive categories such as "mushroom-shaped" herniations. This variability leads to mixed cohorts, inconsistent exclusion criteria, and non-comparable outcome measures. Consequently, evidence derived from trials that group protrusions and extrusions together, or that define "uncontained" lesions ambiguously, cannot be directly extrapolated to patients with extruded discs (2, 9, 14).

The natural history of extruded discs also influences therapeutic decision-making. Extruded and sequestered fragments typically elicit a robust inflammatory response that is paradoxically associated with higher rates of spontaneous regression (13, 15, 16). Although many patients improve with conservative care, a substantial subset experiences persistent radicular pain or develops neurological deficits (3, 4, 6, 7, 15). These cases are traditionally referred for surgical discectomy, which has demonstrated excellent outcomes in extruded herniations. As a result, clinical practice has evolved toward a dichotomy in which extruded discs are directed to surgery, whereas protrusions are more often considered for percutaneous intradiscal procedures. Importantly, this pattern appears to reflect historical surgical outcomes rather than absolute contraindications to intradiscal interventions in extruded discs (4, 6, 7).

Within this context, we define the scope of "intradiscal procedures" as needle-based, percutaneous techniques performed under fluoroscopic or CT guidance. Specifically, we focus on PLDD and T-PLDD (8), radiofrequency nucleoplasty (coblation) (9, 17), oxygen-ozone (O_2_-O_3_) discolysis (13, 18-21), gelified ethanol injection (10, 17, 22), and mechanical intradiscal decompression devices (11, 23). Epidural steroid injections are excluded because they are perineural rather than intradiscal (24), and endoscopic discectomy is also excluded because it represents a surgical rather than a percutaneous needle-based approach.

Other intradiscal modalities, including intradiscal electrothermal therapy, intradiscal biacuplasty, platelet-rich plasma (PRP), cell-based therapies, and other biologic approaches, were not included in the main synthesis. This exclusion reflects the scope of the present review rather than solely the absence of extrusion-specific trials. Our aim was to evaluate percutaneous intradiscal procedures primarily used or debated for lumbar disc herniation with radicular symptoms, particularly interventions intended to reduce disc volume, chemically modify herniated material, or decompress the affected nerve root. By contrast, biacuplasty, intradiscal PRP, cell-based therapies, and similar regenerative or annular pain procedures have been studied predominantly in discogenic axial low back pain or degenerative disc disease rather than in extruded lumbar disc herniation with radiculopathy (25, 26). These modalities therefore fall outside the primary question of this review and were not synthesized in detail.

Across studies, outcomes in extruded cohorts are typically assessed using patient-reported pain and function scores, crossover to surgery, radiologic changes in disc morphology, and the incidence of procedure-related complications. Patient selection criteria recurring across published reports emphasize non-sequestered continuity with the parent disc, absence of major neurological deficits or red flags such as cauda equina syndrome (CES), clinicoradiologic correlation, failure of adequate conservative management, and technical feasibility based on disc anatomy. These definitions and boundaries establish the framework for evaluating the safety and potential utility of intradiscal procedures in extruded lumbar discs.

## 2. Evidence Acquisition

We conducted a structured narrative review to evaluate the safety, efficacy signals, and clinical applicability of needle-based intradiscal interventions for extruded, non-sequestered lumbar disc herniations. The review was designed as a narrative evidence synthesis rather than a systematic review or meta-analysis. Therefore, no pooled effect estimates were calculated; instead, emphasis was placed on morphology-specific interpretation, safety reporting, and clinical relevance within guideline-based care pathways.

### 2.1. Information Sources and Search Strategy

A focused literature search was performed in PubMed/MEDLINE, Embase, Scopus, and the Cochrane Library from database inception to September 2025. Additional targeted searches were conducted in Google Scholar and by screening the reference lists of key articles and relevant reviews. Manufacturer and technical documents were consulted only when needed to clarify device-specific selection criteria, exclusions, or contraindications, particularly for mechanical decompression systems.

The PubMed search strategy combined morphology-related terms with intervention-specific terms as follows:

(lumbar disc herniation OR lumbar disk herniation OR lumbar radiculopathy OR sciatica) AND (extruded OR extrusion OR uncontained OR non-contained OR sequestrated OR sequestered OR free fragment) AND (intradiscal OR intra-discal OR percutaneous) AND (PLDD OR targeted PLDD OR laser disc decompression OR nucleoplasty OR coblation OR oxygen-ozone OR ozone OR O_2_-O_3_ OR gelified ethanol OR Discogel OR mechanical decompression OR Dekompressor OR automated percutaneous lumbar discectomy)

Comparable search terms were adapted for the other databases. No language restriction was applied at the search stage; however, only articles with an English full text or sufficient English abstract data for outcome extraction were included in the synthesis.

### 2.2. Study Selection

Records were screened in two stages. First, titles and abstracts were reviewed to identify articles addressing lumbar disc herniation and intradiscal or percutaneous disc-directed interventions. Second, potentially relevant full texts were assessed for eligibility based on the predefined scope of this review. Studies were eligible if they reported clinical outcomes in adult patients undergoing one of the following intradiscal interventions: PLDD or T-PLDD, radiofrequency nucleoplasty/coblation, oxygen-ozone discolysis, gelified ethanol injection, or mechanical intradiscal decompression devices.

We prioritized studies that explicitly included extruded or uncontained lumbar disc herniations, particularly when non-sequestered continuity with the parent disc was described. Studies with mixed morphology cohorts were included only when extruded cases were reported separately or clearly permitted within the inclusion criteria. When morphology was not clearly stratified, the study was retained only as indirect evidence and was interpreted cautiously.

Exclusion criteria were pediatric populations; non-lumbar disc herniations unless lumbar data were separately extractable; purely basic science or animal studies without direct clinical correlation; case reports or very small case series with fewer than 10 patients; studies limited to epidural or perineural injections; studies focused on endoscopic, microscopic, or open discectomy; and intradiscal therapies primarily designed for discogenic axial low back pain, annular nociception, or regenerative treatment of degenerative disc disease rather than lumbar disc herniation with radiculopathy.

### 2.3. Evidence Yield and Study Types

Because this article was designed as a structured narrative review rather than a systematic review, a PRISMA-style flow diagram was not generated. However, to improve transparency in response to reviewer feedback, we performed a retrospective screening-yield audit using sources for which record counts could be reconstructed during revision. The reconstructable search yield included 177 records in total: 55 from PubMed/MEDLINE, 4 from the Cochrane Library, 100 records screened from the first 20 results of each of five modality-specific Google Scholar searches, and 18 additional candidate records identified through reference-list and citation checking of key studies and reviews. After removal of duplicates and clearly irrelevant records, 82 titles and abstracts were screened for relevance to lumbar disc herniation, disc morphology, intradiscal or disc-directed intervention, clinical outcomes, and safety reporting. Of these, 42 full-text articles or technical documents were assessed in detail. Finally, 16 study-level entries were retained in the evidence map. To avoid assigning indirect or background evidence the same interpretive weight as direct extrusion-specific evidence, the evidence map was explicitly divided into two sections. Table 1A in Supplementary File summarizes the core evidence base, including direct extrusion-specific studies, uncontained-disc studies, or clinically relevant studies in which extruded or uncontained morphology was central to the clinical question. Table 1B in Supplementary File summarizes supportive, indirect, background, or evidence-against-use studies, including mixed-morphology cohorts, intraforaminal or perineural studies retained only for contextual comparison, discogenic-pain cohorts, contained-disc mechanical decompression evidence, and mechanistic or technical background literature. Studies were excluded from the evidence map if they focused exclusively on epidural or perineural injections without relevance to intradiscal decision-making, endoscopic or open surgical discectomy, discogenic axial low back pain without herniation-related radiculopathy, regenerative treatment of degenerative disc disease, pediatric or non-lumbar populations, case reports or very small series, or if disc morphology and outcomes were not sufficiently relevant to the present review question.

**Table 1.** Practical Clinical Positioning of Intradiscal Procedures in Extruded Non-Sequestered Lumbar Disc Herniation ^a^

Technique	Practical Answer in Extruded Non-sequestered LDH	Evidence Basis/Directness	Best Quantitative Signal	Main Safety Concern/Contraindication	Bottom-Line Clinical Position
**Targeted PLDD/standard PLDD**	Yes, but mainly when the extruded component is non-sequestered, anatomically targetable, and not calcified. Standard PLDD is less certain because it relies more on intradiscal pressure reduction.	Direct but limited evidence for T-PLDD; mostly indirect or contained-disc evidence for standard PLDD.	In the T-PLDD series, modified MacNab success rates were 80.0%, 88.0%, 92.0%, and 92.0% at 1, 3, 6, and 12 months, respectively (8).	No serious complications or neurological sequelae were reported in the T-PLDD cohort (8). Avoid sequestered fragments, cauda equina syndrome, progressive motor deficit, and heavily calcified discs.	Most extrusion-oriented laser option; possible in selected cases, but supported by small-series evidence only.
**Radiofrequency nucleoplasty/coblation**	Possible in carefully selected uncontained or extruded non-sequestered discs, but patients should be counseled that response is variable and less predictable than in contained protrusions.	Direct but limited evidence from an uncontained-LDH cohort.	Radiating-pain NRS decreased from 9.0 ± 1.2 to 1.4 ± 2.0; excellent/good MacNab outcomes occurred in 29/41 patients (70.7%) (9).	No major complications were reported; 3/41 patients (7.3%) required subsequent surgery for persistent radiating pain (9). Avoid sequestered fragments and urgent surgical presentations.	Feasible and apparently safe in selected patients; best framed as a cautious intermediate option, not a surgical substitute.
**Oxygen-ozone discolysis**	Reasonable to discuss in selected non-sequestered extrusions without red flags, especially when the goal is a low-morbidity biologically active option; extrusion-specific controlled data remain limited.	Mostly indirect mixed-morphology clinical evidence, supported by biological plausibility for macrophage-mediated resorption of exposed nucleus pulposus.	Large clinical experience and mixed-cohort trials support benefit in LDH, but extrusion-specific subgroup estimates are generally unavailable. Bonetti et al. reported complete remission in 64/86 patients with disc disease (74.4%) after oxygen-ozone infiltration (21).	Overall morbidity is low in published series, but morphology-specific complication rates are inconsistently reported (18, 20). Use strict image guidance and appropriate concentration/volume.	Biologically plausible; should be presented as low-certainty, morphology-indirect evidence rather than proven extrusion-specific efficacy.
**Gelified ethanol/Discogel**	Possible when the lesion is non-sequestered and continuity is preserved; less dependent on pure pressure reduction than mechanical aspiration techniques.	Partly direct but mostly mixed-morphology observational evidence; some cohorts included non-contained herniations while excluding free fragments.	Bellini et al. reported significant improvement in 62/73 lumbar patients (85%) (10). Latka et al. reported 48% COMI improvement and 54% VAS reduction at 1 year in the radicular-leg-pain cohort (12).	Bellini et al. reported leakage in 19 patients without clinical side effects (10). Avoid sequestered fragments, severe calcification, and urgent surgical indications.	Reasonable investigational/intermediate option in selected non-sequestered extrusions; evidence remains mainly observational.
**Mechanical decompression devices/APLD/Dekompressor**	No. These techniques should generally be avoided in extruded or uncontained discs because the displaced fragment is not reliably accessible by intradiscal aspiration; device technical guidance supports a contained-disc selection framework and excludes free fragments.	Evidence largely concerns selected contained herniations; available evidence does not establish benefit in extruded or sequestered lesions.	No consistent quantitative benefit is established for extruded lesions. Systematic review and clinical experience support only weak/limited or selected benefit for mechanical decompression in contained herniations (11, 32, 33).	Safety data mainly come from selected contained-disc populations. Free fragments are excluded in device technical guidance, and extrapolation to extruded or sequestered lesions is unsupported (23).	Avoid in extruded or uncontained lesions; evidence and manufacturer technical guidance primarily support selected contained herniations, with free fragments excluded (11, 23, 32, 33).

^a^ This table is intended to support clinical interpretation, not to provide graded treatment recommendations. Evidence directness reflects whether studies explicitly included extruded or uncontained lumbar disc herniations, whether sequestered fragments were excluded or separately identifiable, and whether outcomes were reported for the target morphology. Findings from mixed protrusion/extrusion cohorts without subgroup reporting should be interpreted as indirect. All modalities should be considered only within a guideline-concordant pathway after conservative care and in the absence of cauda equina syndrome, progressive motor deficit, or other urgent surgical indications. Citation numbers correspond to the revised manuscript reference list after addition of the NICE guideline, SPORT trial, and Stryker Disc Dekompressor technical guide. Abbreviations: LDH, lumbar disc herniation; PLDD, percutaneous laser disc decompression; T-PLDD, targeted PLDD; RF, radiofrequency; NRS, numeric rating scale; VAS, visual analog scale; COMI, Core Outcome Measures Index; APLD, automated percutaneous lumbar discectomy.

### 2.4. Handling of Disc Morphology

Because the terms "extrusion," "uncontained herniation," "ruptured disc," and "sequestration" are used inconsistently across the literature, disc morphology was extracted as a key interpretive variable. For each clinical study, we attempted to determine whether extrusions were defined according to formal imaging criteria, whether sequestered fragments were excluded, and whether the reported outcomes applied specifically to extruded discs or to mixed protrusion/extrusion cohorts.

For the purpose of this review, direct evidence was defined as evidence derived from cohorts explicitly including extruded, non-sequestered discs with preserved continuity to the parent disc. Indirect evidence was defined as evidence derived from mixed cohorts, contained-disc cohorts with limited extrusion inclusion, or studies in which extruded morphology was not analyzed separately.

### 2.5. Outcomes of Interest

The primary outcomes of interest were leg-pain intensity measured by Visual Analog Scale (VAS) or Numeric Rating Scale (NRS), functional improvement measured by the Oswestry Disability Index (ODI) or comparable functional scores, patient-reported global outcomes such as MacNab criteria, and the need for subsequent surgical discectomy after intradiscal treatment. Secondary outcomes included radiologic regression of the herniated fragment, changes in disc morphology on MRI, time to clinical improvement, and procedure-related adverse events, including neurological injury, discitis or infection, chemical neuritis, bleeding or hematoma, and device- or energy-related complications. When reported, we also extracted patient- and anatomy-level variables that may influence treatment response, including age, sex, body mass index, smoking status, duration of symptoms, disc height loss, calcification, single- versus multi-level disease, baseline neurological status, and follow-up duration.

### 2.6. Evidence Appraisal and Synthesis

A formal risk-of-bias assessment was not performed because the manuscript was designed as a narrative review and the available literature was highly heterogeneous in design, morphology definitions, and outcome reporting. Nevertheless, the quality and directness of the evidence were considered qualitatively. Evidence was interpreted according to study design, sample size, whether extruded discs were clearly defined, whether sequestered fragments were excluded, follow-up duration, completeness of outcome reporting, and clarity of adverse-event reporting.

For each intervention, the strength of evidence was categorized descriptively as direct but limited evidence, indirect or mixed-morphology evidence, mechanistic or biological plausibility with limited clinical confirmation, or evidence against use. This approach was used to avoid overstating conclusions when available studies were small, retrospective, or not specific to extruded, non-sequestered discs.

### 2.7. Approach to Synthesis

Given the small number of extrusion-specific studies and heterogeneity in outcomes, no meta-analysis was performed. Instead, findings were synthesized qualitatively by intervention. Whenever available, representative quantitative outcomes were extracted, including VAS/NRS changes, ODI changes, MacNab success rates, complication rates, and crossover-to-surgery rates. When only mixed cohorts were available, findings were explicitly labeled as indirect evidence and were not assumed to apply fully to extruded discs.

All interpretations were anchored to guideline-based care pathways. Intradiscal interventions were therefore considered only as selective, non-first-line options after adequate conservative therapy and in the absence of CES, rapidly progressive motor deficit, or other urgent surgical indications.

## 3. Results

### 3.1. Guideline-Anchored Treatment Pathway

International guidelines consistently prioritize a stepwise, conservative-first approach to lumbar disc herniation with radiculopathy in the absence of red flags or urgent neurological indications. Conservative management generally includes patient education, activity modification, exercise-based rehabilitation or physical therapy, and analgesic or anti-inflammatory medication when appropriate (3-6). Epidural injection of local anesthetic and steroid may be considered in selected patients with acute severe sciatica; however, it is perineural rather than intradiscal and therefore remains outside the main scope of this review (5, 6).

Surgical referral is recommended when cauda equina syndrome, rapidly progressive or functionally significant motor deficit, or other red-flag features are present. In patients with persistent disabling sciatica despite adequate non-surgical treatment and concordant imaging findings, surgical decompression or discectomy remains an established treatment option because it directly addresses nerve-root compression and has been evaluated against nonoperative care in lumbar disc herniation (4, 6, 7). Endoscopic discectomy, although less invasive than open or microscopic discectomy, remains a surgical decompressive procedure and is not considered an intradiscal needle-based intervention for the purposes of this review.

With respect to intradiscal, needle-based interventions, major guidelines and guideline reviews provide limited direction and do not offer strong extrusion-specific recommendations. This caution reflects the low certainty of evidence, heterogeneous study designs, mixed protrusion/extrusion cohorts, inconsistent morphology definitions, and limited high-quality data focused specifically on extruded, non-sequestered lumbar disc herniation (3-6). Therefore, any consideration of intradiscal therapy in this setting should be framed as an author-synthesized, evidence-informed decision pathway rather than as a formal guideline recommendation.

Within this framework, guideline-concordant conservative management should first be instituted in the absence of red flags. Patients should then be reassessed after an appropriate conservative interval. If pain and disability remain substantial, but there is no cauda equina syndrome, no rapidly progressive weakness, and imaging demonstrates an extruded yet non-sequestered fragment that correlates with symptoms, a selective intradiscal procedure may be discussed as a non-first-line intermediate option. This discussion should include realistic expectations, the low certainty and indirectness of much of the available evidence, the possibility of delayed or incomplete relief, and a clear contingency plan for timely surgical referral if pain persists or neurological status worsens (3-7).

If intradiscal therapy is pursued within this author-synthesized framework, patient selection and technique are paramount. Candidates should demonstrate: 1) preserved continuity between the extruded fragment and the parent disc; 2) absence of sequestration; 3) concordant radicular symptoms and imaging; 4) failure of a reasonable conservative trial; 5) absence of cauda equina syndrome, progressive motor deficit, or other urgent surgical indications; and 6) anatomical feasibility for safe needle access and targeted energy or injectate delivery. Procedure choice should then be individualized according to mechanism–morphology fit and evidence directness, with modality-specific evidence summarized separately in the following sections, Table 1, and Table 1 in the Supplementary File.

Finally, outcomes should be evaluated against prespecified fail-safe criteria. An inadequate response after a reasonable convalescence, recurrent disabling radicular pain, or any neurological deterioration should prompt expedited surgical reassessment. Framing intradiscal interventions in this way, as selective, non-first-line intermediate options rather than replacements for guideline-supported conservative care or surgical discectomy, keeps the manuscript aligned with guideline principles while acknowledging the limited and heterogeneous evidence available for extruded, non-sequestered lumbar disc herniation (3-7).

**Figure 1.**
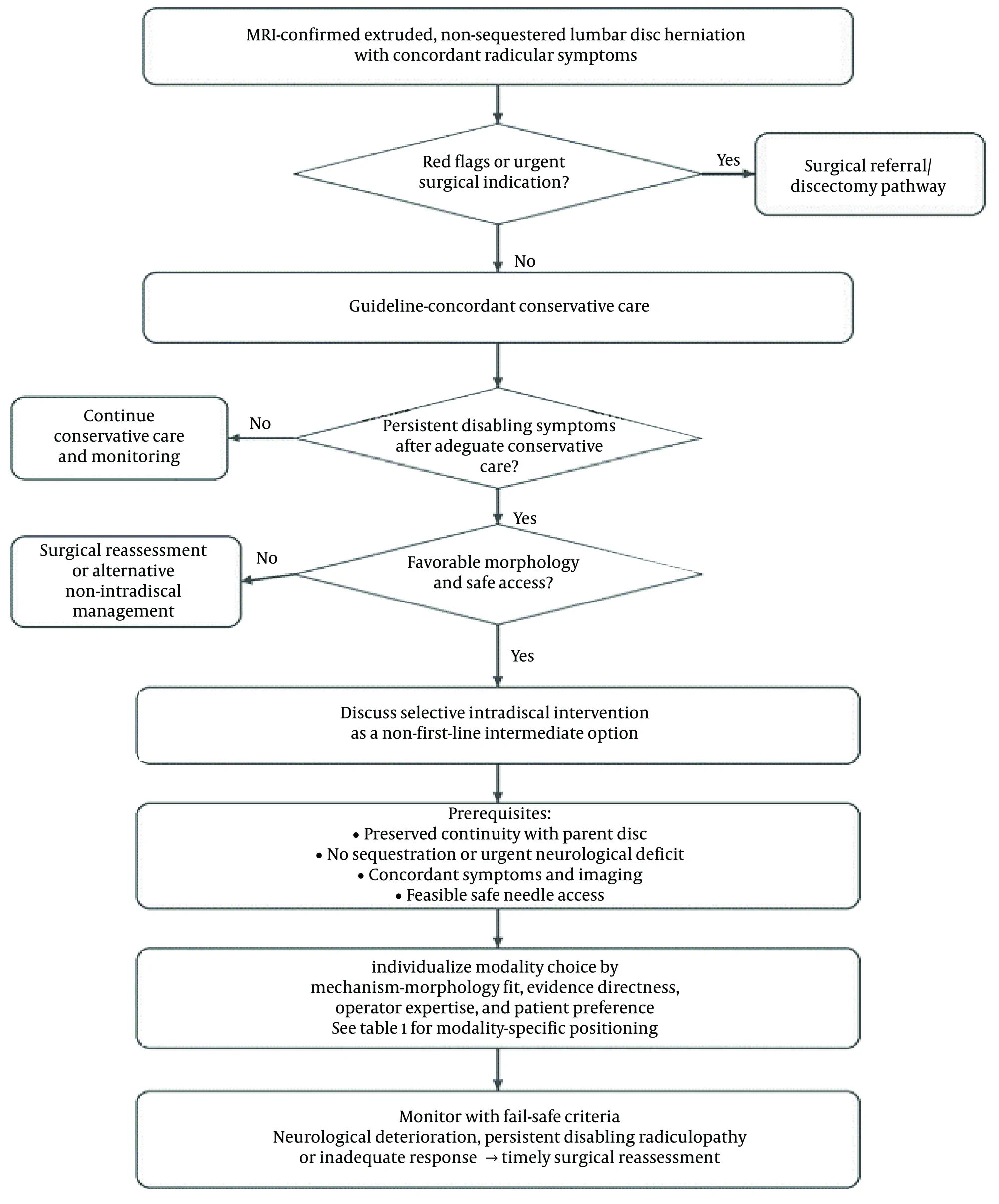
Author-synthesized decision-support framework for considering intradiscal intervention in extruded non-sequestered lumbar disc herniation. This framework is intended to support clinical interpretation and does not represent a formal guideline or graded treatment recommendation. The conservative-care and urgent-surgical-referral steps reflect guideline-aligned principles, whereas the intermediate intradiscal step represents an author-synthesized, evidence-informed option for carefully selected patients. Conservative care remains first-line in the absence of red flags, while urgent neurological indications require timely surgical referral. Intradiscal intervention is presented only as a selective, non-first-line intermediate option in patients with preserved fragment continuity, absence of sequestration, concordant symptoms and imaging, and feasible safe access. Modality choice should be individualized according to mechanism-morphology fit, evidence directness, operator expertise, and patient preference; modality-specific positioning is summarized in Table 1, and the separated study-level evidence map is provided in Table 1 in Supplementary File.

This author-synthesized, guideline-anchored decision-support framework is summarized in Figure 1.

### 3.2. Patient- and Anatomy-Level Modifiers of Response

Several clinical and anatomical variables are likely to influence outcomes after intradiscal interventions in extruded lumbar discs, but they are inconsistently reported across the available literature. These include age, sex, Body Mass Index, smoking status, duration of symptoms, baseline neurological status, degree of disc height loss, calcification, fragment migration, and single- versus multi-level disease. Among these, morphology-related factors appear most immediately relevant to procedural feasibility: preserved continuity with the parent disc, absence of sequestration, absence of severe calcification, and an access trajectory that allows safe delivery of energy or injectate to the intended target. Greater disc collapse, advanced degeneration, or multi-level disease may reduce the likelihood of a predictable response and complicate attribution of symptoms to a single treated level. Because most studies did not report these variables in a standardized manner, the present review could not perform subgroup comparisons based on these modifiers. Accordingly, these factors are treated as selection considerations rather than evidence-based predictors of success.

### 3.3. Percutaneous Laser Disc Decompression

PLDD was first introduced in the late 1980s as a minimally invasive alternative to open discectomy. The original rationale was straightforward: delivery of laser energy into the nucleus pulposus would vaporize a small volume of tissue, thereby reducing intradiscal pressure and indirectly decompressing the affected nerve root. Over time, refinements in laser technology and technique led to the development of T-PLDD, which allows more precise positioning of the laser fiber toward the extruded portion of the disc rather than relying solely on intranuclear pressure reduction (8).

The potential role of PLDD in extruded discs is more controversial than in contained protrusions. Classic intradiscal decompression relies on the integrity of the annulus to transmit pressure reduction, which is disrupted in uncontained herniations. Nevertheless, small clinical series have included patients with extruded but non-sequestered herniations and have reported improvements in pain and function, suggesting that PLDD is not necessarily contraindicated in all non-sequestered extrusions. In these cases, the mechanism may extend beyond simple volume reduction, involving thermal shrinkage of residual annular tissue and modulation of local nociceptive pathways (8).

T-PLDD represents an evolution intended to address this limitation. By directing the laser fiber into the neck of the extrusion or toward the protruding fragment itself, T-PLDD aims to ablate part of the herniated component directly, rather than depending on intradiscal pressure gradients alone. Reports of T-PLDD in extruded herniations, particularly at the L5-S1 level, suggest that with proper selection (non-sequestered continuity, absence of calcification, and no progressive neurological deficit), outcomes may approach those observed in contained lesions (8).

The most directly relevant evidence for extruded, non-sequestered discs comes from the T-PLDD series by Zhao et al., in which modified MacNab success rates increased from 80.0% at 1 month to 92.0% at 6 and 12 months. No serious complications or neurological sequelae were reported in that cohort. Although these findings support technical feasibility and a favorable early safety profile, the evidence remains limited by the small sample size and lack of randomized comparison (8).

In the limited extruded-disc cohorts available, the reported safety profile appears acceptable when strict selection criteria are applied. The most common adverse events described are transient radicular irritation and localized postprocedural pain, whereas serious neurological injury was not reported in the key T-PLDD series. Importantly, contraindications such as sequestered fragments, CES, rapidly progressive motor deficits, or heavily calcified discs must be respected, as PLDD is unlikely to provide benefit and may expose the patient to unnecessary risk in these contexts (8).

In summary, the evidence for PLDD in extruded lumbar discs remains limited and should be interpreted separately for standard PLDD and T-PLDD. Standard PLDD is best established for contained protrusions, whereas T-PLDD provides more directly relevant, but still low-certainty, evidence for extruded, non-sequestered herniations. The available data suggest that extrusion alone should not be viewed as a universal contraindication when continuity is preserved and the extruded component is technically targetable. However, these findings are based on small cohorts and should be applied cautiously, particularly in the absence of randomized extrusion-specific comparisons (8).

### 3.4. Radiofrequency Nucleoplasty

Radiofrequency nucleoplasty, also known as coblation, was introduced in the mid-1990s as a percutaneous technique that uses radiofrequency energy to ablate nucleus pulposus tissue at relatively low temperatures. By creating small plasma channels within the nucleus, nucleoplasty aims to reduce intradiscal volume and pressure while minimizing collateral thermal damage compared with earlier high-temperature methods (9, 17).

The role of nucleoplasty in extruded lumbar discs is less straightforward than in contained protrusions. Because the therapeutic mechanism depends largely on lowering intradiscal pressure, an intact annular barrier is generally considered advantageous. In uncontained extrusions, the efficacy of pressure reduction alone is questioned. Nonetheless, clinical reports and small cohorts have included uncontained or extruded but non-sequestered herniations and have reported pain relief and functional improvement in selected patients. These findings suggest that extrusion, when not sequestered, should not automatically be regarded as a contraindication, although the evidence remains limited (9, 17).

The most relevant direct clinical evidence is the retrospective cohort by Choi et al. evaluating nucleoplasty in uncontained lumbar disc herniation. In that study, mean radiating-pain NRS decreased from 9.0 ± 1.2 before treatment to 1.4 ± 2.0 at final follow-up, and excellent or good MacNab outcomes were reported in 29 of 41 patients (70.7%). However, 3 patients (7.3%) required subsequent surgery for persistent radiating pain, underscoring the need for preprocedure counseling regarding possible treatment failure and surgical crossover (9).

In terms of safety, nucleoplasty has demonstrated a low complication rate across both contained and uncontained cohorts. Reported adverse events are typically minor and transient, including postprocedural soreness and occasional radicular irritation. Serious complications such as nerve injury or infection are rare. Still, the importance of strict patient selection cannot be overstated. Cases with sequestered fragments, CES, rapidly progressive neurological deficits, or advanced disc calcification are inappropriate candidates and should proceed directly to surgery if intervention is indicated (9).

Overall, radiofrequency nucleoplasty has direct but limited evidence in uncontained lumbar disc herniation. The available cohort data suggest that clinically meaningful pain reduction is possible in selected patients, with a low reported rate of major complications and a measurable need for surgical crossover in nonresponders. Therefore, nucleoplasty should be considered a feasible but not definitively established option for extruded, non-sequestered discs. Patients should be counseled that the probability of response is variable and that subsequent surgery may still be required (9, 17).

### 3.5. Oxygen-Ozone Discolysis

Oxygen-ozone discolysis emerged in Europe during the early 1990s as a chemical intradiscal therapy aimed at reducing disc volume and modulating the inflammatory cascade associated with herniated discs (20). The procedure involves percutaneous injection of a carefully titrated oxygen-ozone mixture into the disc, most commonly at a concentration between 20 and 30 μg/mL. Beyond its mechanical dehydrating effect on nucleus pulposus tissue, ozone demonstrates notable biochemical activity, including inhibition of proinflammatory cytokines, reduction of oxidative stress through activation of Nrf2 pathways, and promotion of macrophage-mediated phagocytosis of herniated material. These mechanisms are particularly relevant in extruded herniations, in which exposed nucleus tissue provokes a strong immune response and spontaneous regression is more likely (13, 15, 16, 27, 28).

Evidence for ozone therapy in extruded discs remains limited and mostly indirect. Most randomized trials and observational series include mixed populations of protrusions and extrusions, and extrusion-specific subgroup estimates are usually unavailable. Pain relief and functional improvement have been reported after oxygen-ozone treatment in lumbar disc herniation populations, but these findings cannot be assumed to apply fully to extruded, non-sequestered discs (18, 19). The immune-modulating properties of ozone provide a biological rationale for potential benefit in extrusions, where exposed nucleus pulposus may interact more directly with inflammatory and resorptive pathways (13).

Quantitative evidence for oxygen-ozone therapy is supportive but mostly indirect for extruded discs. Muto et al. reported extensive clinical experience with oxygen-ozone injection in lumbar disc herniation, but morphology-specific outcomes for extruded non-sequestered lesions were not separately extractable. Ozcan et al. reported a decrease in pain score from 6.97 ± 0.11 at baseline to 4.25 ± 0.19 at 1 month and 5.22 ± 0.20 at 24 months, although the cohort was not extrusion-specific. Similarly, randomized and meta-analytic data support benefit and low morbidity in lumbar disc herniation populations, but most studies do not provide extruded-disc subgroup estimates (18-21).

The reported safety profile of oxygen-ozone discolysis appears acceptable when the procedure is performed under strict imaging guidance and with adherence to recommended concentrations and volumes. Serious neurological or infectious complications have been uncommon in available clinical reports, and most reported adverse events are transient (18, 19). Careful technique is essential to avoid inadvertent intravascular or perineural injection. Patients with sequestered fragments, cauda equina syndrome, or progressive motor deficits should not be treated as routine intradiscal candidates and require guideline-concordant surgical assessment when clinically indicated (3, 4, 6).

Taken together, oxygen-ozone therapy has a biological rationale for extruded herniations, particularly because immune exposure of nucleus pulposus may facilitate resorption. However, most clinical evidence remains indirect, derived from mixed-morphology cohorts or studies in which extrusion-specific outcomes were not separately reported. Accordingly, ozone should not be presented as definitively effective for extruded discs, but rather as a biologically plausible option with low reported morbidity in selected reports. Higher-quality extrusion-specific trials are needed before stronger recommendations can be made (13, 18-21, 27).

### 3.6. Gelified Ethanol

Gelified ethanol, commercially known as Discogel, was introduced in 2007 as a chemonucleolysis agent designed to combine the dehydrating properties of ethanol with improved safety and precision. The gel matrix contains radiopaque tungsten, allowing fluoroscopic control during injection, and its increased viscosity reduces the risk of uncontrolled diffusion beyond the disc. By inducing coagulation necrosis and dehydration of nucleus pulposus tissue, gelified ethanol reduces intradiscal pressure while simultaneously exerting a chemical sclerosing effect that can stabilize the degenerated disc environment (12, 17, 22, 29).

Clinical studies of gelified ethanol have included patients with both protruded and extruded herniations. In mixed or partly uncontained cohorts, pain reduction, functional improvement, and radiographic evidence of herniation shrinkage have been reported at follow-up intervals ranging from months to several years. Importantly, most protocols exclude sequestered free fragments, which lack continuity with the parent disc and are unlikely to respond to an intradiscal agent. Within the boundaries of this selection, the available data suggest that extrusion is not necessarily an absolute contraindication to gelified ethanol therapy, although extrusion-specific efficacy remains insufficiently established (10).

Representative quantitative data are encouraging but largely derived from mixed or partly uncontained cohorts. Bellini et al. reported significant clinical improvement in 62 of 73 lumbar patients (85%), defined by meaningful VAS and ODI reduction, and noted contrast leakage in 19 patients without clinical side effects. In the prospective cohort by Latka et al., the radicular-leg-pain subgroup showed 48% improvement in COMI and 54% reduction in VAS at 1 year, with 81% of patients analgesic-free at 6 months. These findings support potential clinical utility but do not establish extrusion-specific efficacy (10, 12). Marcia et al. similarly reported improvement in symptomatic disc herniation, with VAS decreasing from 8 to 3 and ODI from 51 to 15 at 12 months, although morphology-specific outcomes for extruded discs were not isolated (30).

The safety profile of gelified ethanol is generally reassuring in published clinical series. Serious complications such as infection, neurological injury, or chemical neuritis are rarely reported, whereas mild transient back or radicular pain may occur after injection (10, 17, 22, 30, 31). Sequestered free fragments should generally be excluded because intradiscal chemonucleolysis depends on continuity with the parent disc and feasible intradiscal delivery (10). Patients with cauda equina syndrome or progressive neurological deficit require guideline-concordant surgical assessment rather than routine intradiscal treatment (3, 4, 6).

In summary, gelified ethanol appears to be a potentially useful intradiscal option for selected lumbar disc herniations, including some uncontained or non-sequestered lesions. Nevertheless, most available studies include mixed morphologies and do not consistently isolate extruded-disc outcomes. The current evidence is therefore best regarded as observational and partly indirect. Gelified ethanol may be considered when continuity with the parent disc is preserved and surgical indications are absent, but its role in extruded discs remains investigational rather than established (10, 12, 22, 30, 31).

### 3.7. Mechanical Decompression Devices

Mechanical decompression techniques, including automated percutaneous lumbar discectomy (APLD) and later devices such as the Dekompressor, were developed to mechanically remove nucleus pulposus material through a percutaneous approach. The principle is direct aspiration or mechanical extraction of accessible disc tissue, thereby reducing intradiscal pressure and potentially relieving nerve-root irritation. These methods were attractive in the 1990s and early 2000s as minimally invasive alternatives to surgery, particularly for selected contained herniations (11, 23, 32).

The role of mechanical decompression in extruded discs is highly limited. Because the therapeutic effect depends on accessing and removing nuclear material that remains mechanically reachable from within the disc space, these devices are best suited to contained herniations rather than displaced extruded fragments. In extruded or uncontained herniations, particularly when a fragment extends beyond the annulus or has limited mechanical continuity with the intradiscal nucleus, aspiration-based devices cannot reliably engage or remove the displaced component (32, 33). This interpretation is consistent with the Stryker Disc Dekompressor technical guide, which states that the probe is not appropriate for pain originating from structures other than contained herniated discs, excludes patients with free fragments, and lists MRI findings consistent with contained disc herniation as part of ideal patient selection (23).

Accordingly, the evidence base for mechanical decompression should be interpreted as evidence for contained herniations rather than for extruded lesions. Published reviews and large clinical experiences may support technical feasibility in selected contained discs, but they do not establish benefit in extruded non-sequestered herniations. Device-specific technical guidance further reinforces this contained-disc selection framework and does not support extrapolation to free-fragment, sequestered, or clearly uncontained lesions (11, 23, 32, 33).

Safety profiles for mechanical decompression in selected contained herniations have generally been acceptable, with low rates of infection, bleeding, or neural injury reported in appropriately selected populations. However, these safety data cannot be directly extrapolated to extruded or uncontained lesions. In this setting, the principal concerns are not only procedural complications but also technical failure, delayed definitive decompression, and the subsequent need for surgery. No consistent extrusion-specific efficacy or safety signal supports routine use of aspiration-based mechanical decompression in extruded lumbar disc herniation (11, 23, 32, 33).

In conclusion, mechanical decompression devices differ from the other modalities reviewed because their mechanism relies on aspiration or extraction of accessible intradiscal nuclear material, whereas extruded fragments are displaced beyond the usual target zone of these devices. The available evidence and manufacturer technical guidance support their use, if at all, mainly in selected contained herniations and do not establish benefit in extruded non-sequestered lesions. Mechanical decompression should therefore be distinguished from modalities with direct or biologically plausible relevance to extruded discs and should not be presented as an expandable option for this population (11, 23, 32, 33).

The practical clinical positioning of each intradiscal modality, integrating evidence directness, representative outcomes, safety signals, and applicability to extruded non-sequestered discs, is summarized in Table 1.

## 4. Conclusions

### 4.1. Comparative Clinical Interpretation

The reviewed interventions should not be interpreted as equivalent members of a single therapeutic class. Their applicability to extruded, non-sequestered discs depends on both the directness of the clinical evidence and the biological or mechanical plausibility of their mechanisms in an uncontained morphology. T-PLDD and radiofrequency nucleoplasty provide the most direct, although still limited, clinical evidence in extruded or uncontained herniations. T-PLDD is conceptually attractive because it attempts to address the extruded component directly rather than relying solely on intranuclear pressure reduction, whereas nucleoplasty provides a decompressive intradiscal approach with reported benefit in uncontained cohorts but also a measurable rate of surgical crossover (8, 9).

Oxygen-ozone discolysis and gelified ethanol occupy a different position. Their mechanisms are not purely mechanical and may be less dependent on intact annular containment than aspiration-based devices. Oxygen-ozone therapy is supported by a plausible biological rationale involving modulation of inflammation and potential facilitation of fragment resorption, but its clinical evidence in extruded discs remains largely indirect because most studies include mixed morphologies or do not report extrusion-specific subgroup outcomes (13, 18-21, 27). Gelified ethanol has encouraging observational data, including cohorts that allowed non-contained lesions or excluded free fragments; however, the evidence remains predominantly mixed-morphology and insufficient to establish a definitive extrusion-specific effect (10, 12, 17, 22, 30, 31).

Mechanical decompression devices represent the clearest exception. Because their mechanism relies on aspiration or extraction of accessible intradiscal nuclear material, they are poorly suited to displaced extruded fragments. The reviewed clinical evidence and device-specific technical guidance support, at most, use in selected contained herniations and do not establish benefit in extruded or sequestered lesions. Therefore, unlike T-PLDD, oxygen-ozone discolysis, nucleoplasty, or gelified ethanol, mechanical decompression should not be considered an expandable option for extruded lumbar disc herniation (11, 17, 23, 32, 33).

Taken together, the available literature supports a cautious middle position: extrusion alone should not be treated as a universal contraindication to all intradiscal procedures, but neither should intradiscal intervention be presented as an established substitute for surgical decompression. Across modalities, the key determinant is not extrusion alone but the combination of fragment continuity, mechanism of action, anatomical feasibility, clinical urgency, and the patient’s willingness to accept uncertain benefit and possible surgical crossover. T-PLDD and nucleoplasty have the most direct but still limited clinical evidence in extruded or uncontained herniations; oxygen-ozone discolysis has the strongest mechanistic rationale for extrusion-related resorption but relies largely on indirect clinical evidence; gelified ethanol has encouraging mixed-morphology data, including partly uncontained-disc cohorts, but lacks robust extrusion-specific trials; and mechanical decompression has evidence against use in this setting. This pragmatic positioning is summarized in , while study-level details and evidence directness are provided in Table 1A and Table 1B in Supplementary File, which separate the core evidence base from supportive, indirect, background, and evidence-against-use studies. This selective and individualized positioning is also consistent with the broader movement toward personalized pain management, in which treatment choice is tailored to patient-level, anatomical, and disease-specific factors rather than applied uniformly across heterogeneous pain conditions (34).

Importantly, this clinical positioning should be interpreted within established guideline-based care rather than as a competing treatment pathway. Conservative management remains the initial standard for most patients without red flags, and surgical decompression remains the reference treatment for cauda equina syndrome, progressive motor deficit, or persistent disabling radiculopathy with concordant imaging. Within this framework, intradiscal procedures should be considered only as selective, non-first-line intermediate options for carefully selected patients who wish to avoid or defer surgery and who have extruded but non-sequestered herniations, preserved continuity with the parent disc, concordant symptoms and imaging, and no urgent surgical indication. This position is not intended to upgrade the level of recommendation for intradiscal procedures, but rather to clarify that extrusion alone, when non-sequestered and technically targetable, should not automatically be interpreted as a universal contraindication to every intradiscal modality (3-7).

Patient selection is therefore the central safety variable. The margin for error is smaller in extruded lesions because the displaced fragment may lie closer to neural structures and because a failed procedure can delay definitive decompression (35). Strict exclusions should include cauda equina syndrome, rapidly progressive or functionally significant motor weakness, sequestered free fragments, severe calcification that precludes safe targeting, active infection, and uncorrected coagulopathy. In all cases, patients should be counseled that the certainty of evidence remains low, that the probability of benefit varies by modality and morphology, and that clinical deterioration should prompt timely surgical referral.

### 4.2. Limitations and Future Directions

This review has several limitations. First, the definition of extruded disc herniation is not uniform across the literature. Some studies classify uncontained but continuous fragments as extrusions, whereas others include or fail to distinguish sequestered fragments. This heterogeneity limits direct comparison across techniques and complicates interpretation of morphology-specific outcomes. Second, many studies combine protruded, extruded, and uncontained discs within the same cohort, making it difficult to extract outcomes specifically for extruded, non-sequestered herniations. Third, the available evidence is dominated by small case series, retrospective cohorts, observational studies, and mixed-population trials. Formal risk-of-bias assessment was not performed because this was a structured narrative rather than a systematic review; therefore, the overall certainty of evidence should be considered low.

Fourth, adverse-event reporting is inconsistent and may underestimate true complication rates. This limitation is particularly relevant in extruded discs, in which the displaced fragment may be closer to neural structures and procedural failure may delay definitive surgical decompression. Fifth, patient- and anatomy-level modifiers such as disc height loss, calcification, migration, symptom duration, smoking status, body mass index, baseline neurological status, and single- versus multi-level disease were inconsistently reported, preventing meaningful subgroup analysis. Finally, publication bias cannot be excluded, especially for emerging intradiscal technologies and commercially driven interventions.

Future studies should be designed specifically around disc morphology. Trials should distinguish protrusion, extrusion, migration, and sequestration using standardized imaging definitions and should explicitly report whether continuity with the parent disc is preserved. Clinically meaningful designs would include randomized or prospective comparative studies evaluating targeted PLDD, oxygen-ozone discolysis, gelified ethanol, or nucleoplasty against continued conservative care, sham-controlled procedures where ethically feasible, or surgical discectomy in patients without urgent neurological indications. Outcomes should include leg-pain VAS or NRS, ODI, MacNab success, MRI-based fragment regression, time to improvement, crossover to surgery, and standardized adverse-event reporting. Until such evidence is available, intradiscal procedures in extruded, non-sequestered discs should be framed as selective and investigational or intermediate options rather than guideline-established replacements for surgery.

### 4.3. Conclusions

Extruded lumbar disc herniation has traditionally been viewed as a predominantly surgical condition, particularly when symptoms are severe or neurological compromise is present. This review suggests that extrusion alone should not be regarded as a universal contraindication to all intradiscal interventions. However, the current evidence supporting intradiscal procedures in extruded, non-sequestered discs remains limited and heterogeneous and is largely derived from small cohorts, retrospective studies, or mixed-morphology populations.

Among the reviewed modalities, targeted PLDD and radiofrequency nucleoplasty provide the most direct but still low-certainty clinical evidence in extruded or uncontained herniations. Oxygen-ozone discolysis and gelified ethanol have biologically plausible mechanisms and encouraging mixed-morphology clinical data, but robust extrusion-specific outcomes remain insufficient. Mechanical decompression devices, in contrast, should not be used in extruded discs because their mechanism, available evidence, and device-related guidance do not support this indication.

Accordingly, intradiscal procedures should be framed as selective, non-first-line intermediate options rather than replacements for guideline-supported conservative care or surgical discectomy. Their consideration should be restricted to carefully selected patients with preserved continuity between the extruded fragment and the parent disc, absence of sequestration, no cauda equina syndrome or progressive motor deficit, concordant clinicoradiologic findings, and clear counseling regarding uncertain benefit and possible surgical crossover. Future extrusion-specific trials using standardized morphology definitions, validated clinical outcomes, imaging follow-up, and rigorous safety reporting are required before these techniques can be incorporated more confidently into formal treatment algorithms.

aapm-16-1-168119-s001.pdf

## Data Availability

The dataset presented in the study is available on request from the corresponding author during submission or after publication.
