# Supplementary material for: From Protrusion to Extrusion: Reassessing the Selective Role of Intradiscal Interventions
Source: Anesth Pain Med. 2026 Feb 28;16(1):e168119. doi: 10.5812/aapm-168119 (PMC13589456; doi:10.5812/aapm-168119)
Supplement: Supplementary file 1 [file aapm-16-1-168119-s001.pdf]

Supplementary Table 1

Study-Level Evidence Map for Intradiscal Interventions in Extruded or Uncontained Lumbar Disc Herniation

Supplementary Table 1A. Core or Closest Clinical Evidence Relevant to Extruded or Uncontained Lumbar Disc Herniation

Ref.	Study	Modality	Design / sample	Morphology and relevance to extrusion	Follow-up	Key outcomes	Safety / crossover	Directness
8	Zhao et al., 2012	T-PLDD	Clinical case series; n=25	Posterolateral extruded but non-sequestered LDH; direct targeting of the extruded component.	1, 3, 6, 12 months	Modified MacNab success rates were 80.0%, 88.0%, 92.0%, and 92.0% at 1, 3, 6, and 12 months, respectively.	No serious complications or neurological sequelae were reported.	Direct but limited
9	Choi et al., 2024	RF nucleoplasty / coblation	Retrospective cohort; 41 analyzed patients	Uncontained LDH; contained herniations excluded; directly relevant to extrusion/uncontained morphology.	Minimum 3 months; final follow-up reported	Radiating-pain NRS decreased from 9.0 ± 1.2 to 1.4 ± 2.0; excellent/good MacNab outcomes occurred in 29/41 patients (70.7%).	No major complications were reported; 3/41 patients underwent subsequent surgery.	Direct but limited
10	Bellini et al., 2015	Gelified ethanol / RGE	Retrospective clinical series; 80 patients; 107 lumbar and 9 cervical treated discs	Contained and non-contained herniations included if PLL integrity was preserved; free isolated fragments were excluded. This is clinically relevant to non-sequestered extrusion, but extrusion-specific outcomes were not isolated.	Clinical follow-up reported; timing varied	Significant improvement was reported in 62/73 lumbar patients (85%) and 6/7 cervical patients (83%), defined by VAS reduction >=4 and ODI reduction >=40%.	Leakage occurred in 19 patients without clinical side effects.	Partly direct; subgroup not isolated

Supplementary Table 1B. Supportive, Indirect, Background, or Evidence-Against-Use Studies

Ref.	Study	Modality	Design / sample	Morphology and relevance to extrusion	Follow-up	Key outcomes	Safety / crossover	Directness
20	Muto et al., 2004	Oxygen-ozone	Large observational experience; n=2,200	Lumbar disc herniation cohort; morphology not stratified into protrusion versus extrusion in extracted data. Retained as broad mixed-morphology ozone evidence.	Variable / not uniformly extractable	Large clinical experience supports feasibility and symptomatic improvement; extrusion-specific outcome unavailable.	Complications not extractable by morphology.	Indirect mixed-morphology
21	Bonetti et al., 2005	Oxygen-ozone vs steroid	Randomized controlled study; disk disease subgroup reported	Lower back pain/disk disease; intraforaminal O2-O3 rather than purely intradiscal; morphology not extrusion-specific. Retained only for contextual comparison.	Short-, medium-, and long-term follow-up	After O2-O3 infiltration, complete remission was reported in 64/86 (74.4%) disk disease patients.	Morphology-specific safety not available in extracted data.	Indirect supportive / perineural-contextual
19	Ozcan et al., 2018	Intradiscal oxygen-ozone	Clinical trial; n=63	Single-level bulging/protrusion cohort; not an extruded-disc cohort.	1, 3, 6, 12, and 24 months	Pain score decreased from 6.97 ± 0.11 at baseline to 4.25 ± 0.19 at 1 month and 5.22 ± 0.20 at 24 months; excellent/good MacNab outcomes occurred in 21/63 patients (33.3%).	No extrusion-specific safety signal available.	Indirect; not extrusion-specific
18	Magalhaes et al., 2012	Ozone therapy	Systematic review/meta-analysis of RCTs	Mixed LDH populations; extrusion-specific outcomes not separately reported.	Varied	Ozone therapy showed positive clinical results and low morbidity overall; no extruded subgroup estimates.	Low morbidity overall; morphology-specific complications unavailable.	Indirect mixed-morphology
13	Erario et al., 2021	Ozone rationale	Mechanistic / conceptual review	Focused on extruded nucleus pulposus herniation and immune-mediated resorption. Retained as biologic plausibility rather than clinical outcome evidence.	Not applicable	Supports biological plausibility of ozone-mediated modulation of resorption and inflammatory response.	Not a clinical safety study.	Supportive mechanistic
22	Theron et al., 2007	Gelified ethanol / RGE	Preliminary clinical study	Lumbar intervertebral disk hernias treated with radiopaque gelified ethanol; morphology-specific extraction limited.	Not extractable from current manuscript data	Reported as foundational preliminary experience for gelified ethanol chemonucleolysis.	Detailed morphology-specific safety not extractable here.	Indirect/supportive
31	Touraine et al., 2015	Ethanol gel chemonucleolysis	Cohort study; n=42	Sciatica refractory to conservative therapy with concordant disc herniation; extrusion subgroup not separately reported.	1 and 3 months	Pain intensity decreased 44% at 1 month and 62.6% at 3 months; physician-rated improvement occurred in 30/42 patients (71.4%) at 1 month and 36/42 patients (85.7%) at 3 months.	Morphology-specific safety not extractable in current data.	Indirect mixed-morphology

Ref.	Study	Modality	Design / sample	Morphology and relevance to extrusion	Follow-up	Key outcomes	Safety / crossover	Directness
12	Latka et al., 2021	Gelified ethanol / Discogel	Prospective observational cohort; n=67; DP n=45, RLP n=22	RLP cohort included protrusion/extrusion; extruded cases not separately reported. DP cohort represents discogenic pain and is indirect for the present review question.	1 year	In the RLP cohort, COMI and VAS decreased by 48% and 54% at 1 year; 81% were analgesic-free at 6 months.	No adverse effects reported.	Indirect mixed-morphology
30	Marcia et al., 2018	Gelified ethanol / Discogel	Clinical cohort; 71 patients; 87 disc herniations	Symptomatic disc herniation after failed conservative treatment; extrusion-specific subgroup not separately reported.	12 months	VAS decreased from 8 to 3 and ODI from 51 to 15 at 12 months; analgesic use decreased from 70.4% to 29.6%.	No symptomatic procedural complications; one asymptomatic perineural extravasation on MDCT.	Indirect mixed-morphology
17	Papadopoulos et al., 2014	Pulsed RF + gelified ethanol	Pilot study; n=11	Chronic discogenic low back pain with provocative discography; not an extruded herniation cohort. Retained only as background for gelified ethanol-based intradiscal treatment.	Not extractable from current manuscript data	Successful outcome occurred in 9/11 patients (81.8%) based on pain relief and satisfaction.	Not extrusion-specific.	Indirect; discogenic pain
11	Singh et al., 2009	Dekompressor / mechanical	Systematic review	Primarily contained-disc evidence; extruded or sequestered lesions generally outside the target population for aspiration-based decompression.	Short- and long-term outcomes reviewed	Dekompressor evidence was rated weak/limited; no consistent evidence supports use in extruded discs.	No extruded-disc safety/efficacy signal identified.	Evidence against use in extrusion
32	Bonaldi, 2003	APLD	Large clinical experience; >1,000 patients	Technique/indication evidence for percutaneous mechanical discectomy; best suited to contained herniations.	Clinical follow-up reported	Supports APLD in selected contained herniations; does not establish benefit in extruded non-sequestered discs.	Extruded-disc adverse events not separately extractable.	Indirect contained-disc evidence
33	Lierz et al., 2009	Dekompressor	Clinical series using Dekompressor under CT control	Percutaneous lumbar discectomy system; extrusion-specific outcomes not established.	Not extractable from current manuscript data	Supports technical feasibility in selected patients but does not provide evidence for extruded lesions.	Not extrusion-specific.	Indirect; not supportive for extrusion

Note. *Supplementary Table 1 separates the evidence map into core or closest clinically relevant evidence (Supplementary Table 1A) and supportive, indirect, background, or evidence-against-use studies (Supplementary Table 1B). This separation is intended to prevent indirect, perineural, discogenic-pain, contained-disc, or mechanistic evidence from being interpreted with the same weight as direct extrusion-specific or uncontained-disc clinical evidence. Morphology-specific outcomes were reported when available; otherwise, the evidence was labeled as indirect. Citation numbers correspond to the revised manuscript reference list after addition of the NICE guideline and SPORT trial. LDH = lumbar disc herniation; RGE = radiopaque gelified ethanol; PLL = posterior longitudinal ligament; RLP = radicular leg pain; DP = discogenic pain; NRS = numeric rating scale; ODI = Oswestry Disability Index; VAS = visual analog scale; COMI = Core Outcome Measures Index; APLD = automated percutaneous lumbar discectomy.*
